# A Cow Case1Presented at the Fourteenth Annual Meeting of the Iowa State Veterinary Medical Association, Des Moines, Iowa, February 11 and 12, 1902.

**Published:** 1902-04

**Authors:** G. P. Statter

**Affiliations:** Sioux City, Iowa


### A COW CASE.[1]

By G. P. Statter, V.S., M.D.V.,
SIOUX CITY, IOWA.

I was called at 9 A. M. to see a Jersey cow, seven years old, heavy milker when fresh, due to calve in two weeks. At last calving she gave birth to twins. The services of a veterinarian were required to assist delivery, following which, according to the attendant's story, she suffered a mild attack of parturient apoplexy.

The history of the present attack was that she had not been out of the stable for two weeks, and had been fed on straw and corn-fodder. The night previous she had not seemed well, had refused her feed, was very uneasy, and kept paddling with her hind feet, but finally laid down and seemed easy. I found her down, and if I had not known that she had not calved I would have at once said that it was an attack of parturient apoplexy. She was lying with her head in the flank, and on straightening the neck and releasing it it would return as before; eyes were dull, the mouth open, and saliva dripping from it; breathing suppressed, with occasional moaning; nothing had passed from the bowels or bladder since the previous day; there was almost complete coma and partial or complete paralysis of deglutition. I carefully gave a capsule containing half a drachm of croton oil, which I could feel pass down, and administered stimulants in the same way. I emptied the bladder and rectum, had her braced up on the sternum, and well clothed. I left a mixture containing nux vomica and ammonium muriate, with instructions to turn over every four hours. Calling again at night, I could see no change. Relieved the bladder and found the rectum empty, though the animal had passed nothing. The next morning the patient was still down, but had a free evacuation of the bowels, which was very fetid. There were signs of returning consciousness to the extent that the cow was able to eat and drink a little. About noon she made an effort to get up, and with a little assistance did so. When I called that night I found her still standing and apparently well. She calved within three weeks without further trouble.

From the history, symptoms, and results of treatment, What should have been the diagnosis?—ante-partum paralysis, parturient apoplexy, or results of improper treatment?

[1] Presented at the Fourteenth Annual Meeting of the Iowa State Veterinary Medical Association, Des Moines, Iowa, February 11 and 12, 1902.

### Discussion.

Dr. Repp said that he was satisfied the case was one of parturient paralysis, although this disease is rare before parturition.

